# Downregulation of FOXP1 Inhibits Cell Proliferation in Hepatocellular Carcinoma by Inducing G1/S Phase Cell Cycle Arrest

**DOI:** 10.3390/ijms17091501

**Published:** 2016-09-08

**Authors:** Xin Wang, Ji Sun, Meiling Cui, Fangyu Zhao, Chao Ge, Taoyang Chen, Ming Yao, Jinjun Li

**Affiliations:** 1 Shanghai Medical College, Fudan University, Shanghai 200032, China; wangxin230423@163.com (X.W.); jisun13@fudan.edu.cn (J.S.); 2 State Key Laboratory of Oncogenes and Related Genes, Shanghai Cancer Institute, Renji Hospital, Shanghai Jiaotong University School of Medicine, 25/Ln 2200, Xietu Road, Shanghai 200032, China; cuimeiling768@163.com (M.C.); fangyuzhao11@163.com (F.Z.); chaoge1127@163.com (C.G.); myao@shsci.org (M.Y.); 3 Pathological Section, Qidong Liver Cancer Institute, Qidong 226200, China; taoyangchen@yeah.net

**Keywords:** FOXP1, proliferation, hepatocellular carcinoma, cell cycle

## Abstract

Forkhead box P1 (FOXP1) belongs to a family of winged-helix transcription factors that are involved in the processes of cellular proliferation, differentiation, metabolism, and longevity. FOXP1 can affect cell proliferation and migratory ability in hepatocellular carcinoma (HCC) in vitro. However, little is known about the mechanism of FOXP1 in the proliferation of HCC cells. This study aimed to further explore the function of FOXP1 on the proliferation of HCC cells as well as the relevant mechanism involved. Western blot analysis, tumor xenograft models, and flow cytometry analysis were performed to elucidate the function of FOXP1 in the regulation of cell proliferation in human HCC. We observed that silencing FOXP1 significantly suppressed the growth ability of HCC cells both in vitro and in vivo. In addition, knockdown of FOXP1 induced G1/S phase arrest, and the expression of total and phosphorylated Rb (active type) as well as the levels of E2F1 were markedly decreased at 24 h; however, other proteins, including cyclin-dependent kinase (CDK) 4 and 6 and cyclin D1 did not show noticeable changes. In conclusion, downregulation of FOXP1 inhibits cell proliferation in hepatocellular carcinoma by inducing G1/S phase cell cycle arrest, and the decrease in phosphorylated Rb is the main contributor to this G1/S phase arrest.

## 1. Introduction

Hepatocellular carcinoma (HCC) is one of the most prevalent malignant tumors, with rising incidence in recent decades [1]. The prognosis of patients diagnosed with HCC remains poor despite progress with surgical and non-surgical therapies. Notably, numerous studies have identified a variety of molecules that are involved in the initiation and progression of HCC [2]. Thus, it is highly desirable to find new biomarkers and molecular targets to develop novel strategies that will be more effective for patients.

Forkhead box P1 (FOXP1) is a member of the forkhead box P subfamily, which consists of four members (FOXP1-4) [3]. The FOXP subfamily belongs to the FOX superfamily, which has a highly conserved “fork-head” or “winged-helix” DNA-binding domain (DBD) [4]. FOX proteins are involved in cell cycle progression, proliferation, and differentiation, as well as in metabolism, senescence, survival, and apoptosis [5]. FOXP1 is widely expressed in normal human tissues and a diverse range of cancers [4,6,7,8,9,10]. A growing body of evidence suggests that FOXP1 plays an important role in tumorigenesis and the progression of several types of solid tumors. FOXP1 has been identified as having diverse functions, and can act as either a tumor suppressor or an oncogene. FOXP1 is overexpressed in diffuse large B-cell lymphoma (DLBCL) and mucosa-associated lymphoid tissue (MALT) lymphoma through a recurrent chromosomal translocation T (3; 14) (p14.1; q32) involving IGH and FOXP1, which is associated with poor prognosis [11,12,13,14,15]. By contrast, the loss of FOXP1 expression has been observed in human glioma [16], prostate cancer [17], and renal cell carcinoma [18], and the loss of FOXP1 in breast cancer is correlated with lower survival rates [19]. High expression of FOXP1 correlates with an aggressively malignant phenotype and may constitute a novel prognostic factor for HCC [20,21]. Downregulation of FOXP1 resulted in the suppression of migration and reduced viability of SMMC-7721 cells as well as an increase in cell apoptosis [22]. However, the action of FOXP1 in the proliferation of HCC cells is unknown.

In the present study, we investigate the effect of FOXP1 on the proliferation of HCC cells and seek to determine the relevant molecular mechanism.

## 2. Results

### 2.1. FOXP1 Is Frequently Upregulated in HCC Tissues

**Figure 1:**
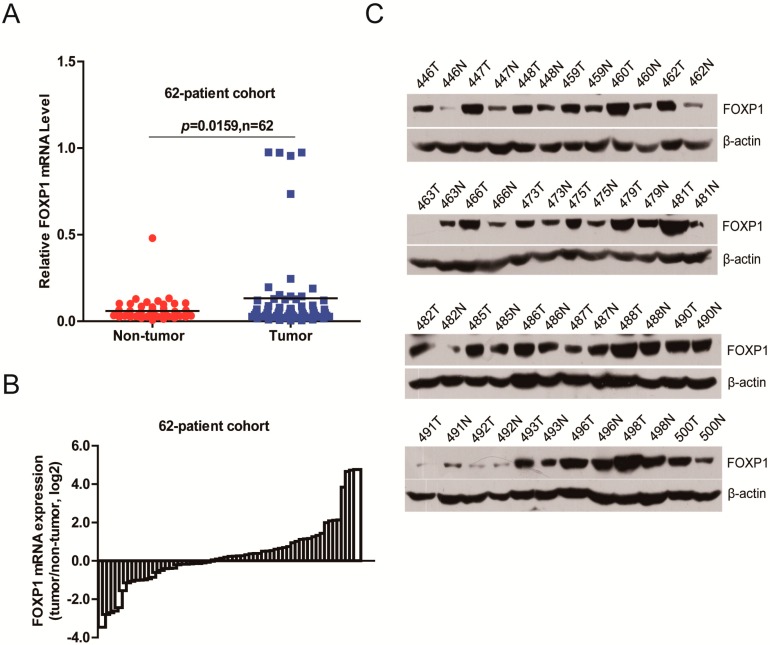
Forkhead box P1 (FOXP1) expression in hepatocellular carcinoma (HCC) clinical samples. (**A**) FOXP1 mRNA levels in the 62-patient cohort; (**B**) The fold change of FOXP1 levels in paired tumor/non-tumorous tissues of the 62-patient cohort; (**C**) FOXP1 protein levels in 24 pairs of HCC tissues (T) and their corresponding adjacent noncancerous tissues (N).

We analyzed the expression levels of FOXP1 in primary HCC samples. The results showed that FOXP1 was significantly upregulated in the cancer tissues of 62 matched HCC/normal samples by qRT-PCR (Figure 1A,B) and 24 matched HCC/normal samples by Western blot analysis (Figure 1C), and a similar result was confirmed in a 50-patient cohort from The Cancer Genome Atlas (TCGA) (Figure S1A,B).

### 2.2. Silencing FOXP1 Inhibits the Growth Ability of HCC Cells in Vitro

**Figure 2:**
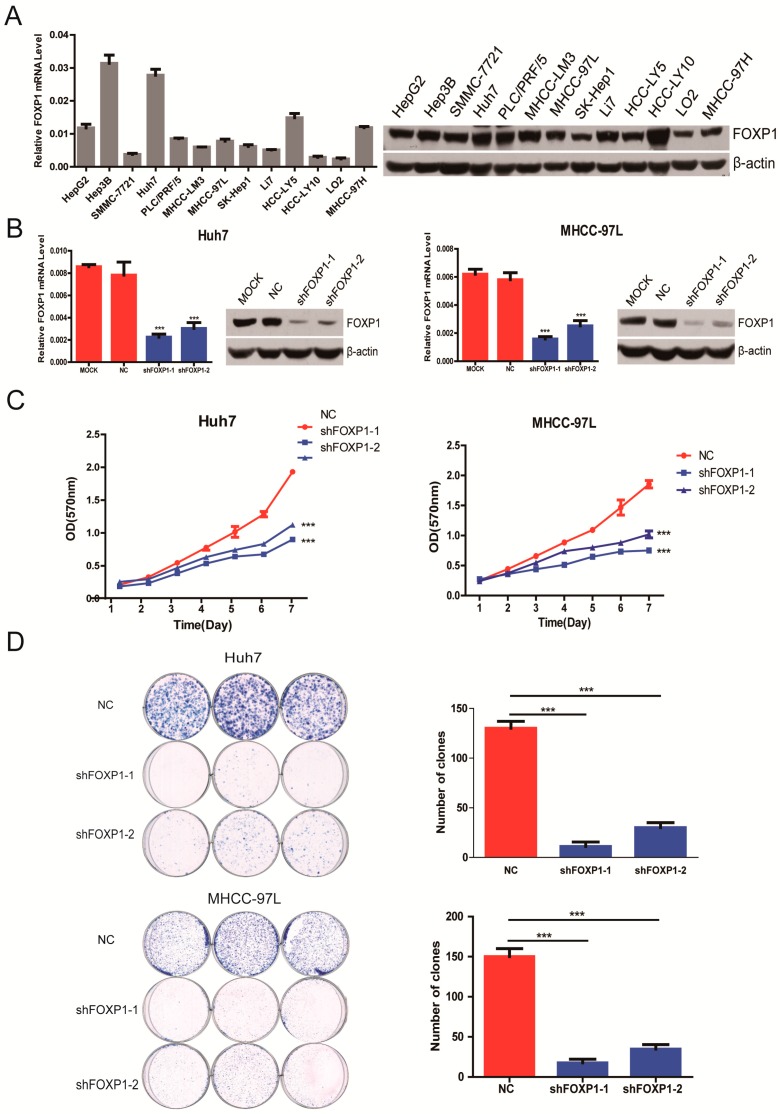
The effect of FOXP1 on the growth ability of HCC cells in vitro. (**A**) FOXP1 mRNA and protein levels in the HCC cell lines; (**B**) FOXP1 mRNA and protein levels in Huh7 and MHCC-97L cells stably transfected with shFOXP1, the scrambled sequence control cells (NC) and untreated cells (MOCK); (**C**) MTT assays for Huh7 and MHCC-97L cells that were stably transfected with either shFOXP1 or NC. *** *p* < 0.001; (**D**) Colony formation assays for Huh7 and MHCC-97L cells that were stably transfected with shFOXP1 or a scrambled sequence control. *** *p* < 0.001.

To proceed with the subsequent research, qRT-PCR and Western blotting were performed to detect the expression of FOXP1 in HCC cell lines. We found that FOXP1 expression was generally high in all of the tested HCC cell lines (Figure 2A). Thus, we selected common Huh7 and MHCC-97L cells, both of which express relative high levels of FOXP1, for our primary study.

To better understand the function of FOXP1 in HCC, four lentiviral vectors expressing various short-hairpin RNAs designed to knockdown FOXP1 (shFOXP1) were stably expressed in Huh7 and MHCC-97L cells. We selected two efficient hairpins (shFOXP1-1 and shFOXP1-2) that sustained more than a 50% reduction of FOXP1 at the mRNA and protein level (Figure 2B). Cell proliferation was detected by the MTT assay for seven days. On the sixth and seventh day, the number of viable cells was significantly decreased in Huh7 and MHCC-97L cells stably expressing shFOXP-1 compared with the controls (Figure 2C). Moreover, the colony formation assays confirmed a similar result (Figure 2D). Therefore, these results suggest that FOXP1 downregulation significantly inhibits HCC cell growth in vitro.

### 2.3. Knockdown of FOXP1 Decreases Tumorigenicity of HCC Cells in Vivo

**Figure 3:**
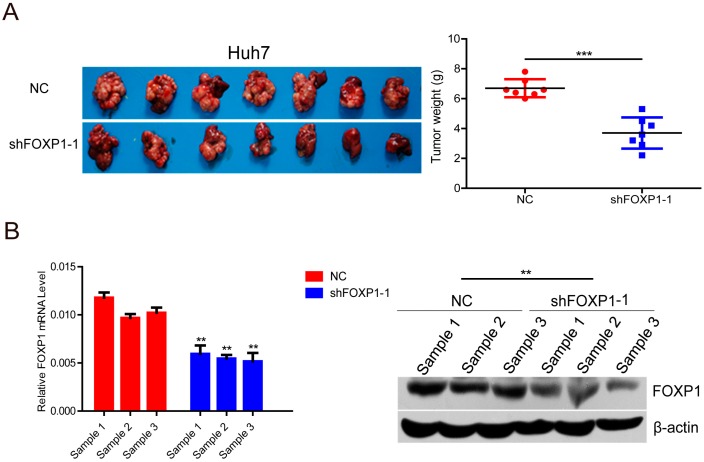
The effect of FOXP1 on the tumorigenicity of HCC cells in vivo. (**A**) Huh7 cells stably expressing shFOXP1-1 were injected orthotopically into nude mice; empty vectors were used as a control. The tumors were removed from the nude mice after four weeks. Representative images are shown along with the weight of the livers with tumors. ** *p* < 0.01; *** *p* < 0.001; (**B**) FOXP1 mRNA and protein levels in the xenograft tumors. ** *p* < 0.01.

To further clarify the effect of endogenous FOXP1 on tumor growth in vivo, Huh7-lenti-shFOXP1 and Huh7-lenti-control cells were orthotopically inoculated in the left hepatic lobe of mice. The tumors in the liver and lung were observed after four weeks. Notably, the tumor weight was remarkably decreased in the shFOXP1-expressing tumor-bearing mice in comparison to the control group (Figure 3A). FOXP1 protein levels in the xenograft tumors were analyzed by qRT-PCR and Western blotting (Figure 3B). These results indicate that FOXP1 plays an important role in tumor formation of HCC and can be a positive regulator of HCC growth.

### 2.4. Downregulation of FOXP1 Induces G1/S Cycle Arrest and Regulates Cell Cycle-Related Proteins in HCC Cells

**Figure 4:**
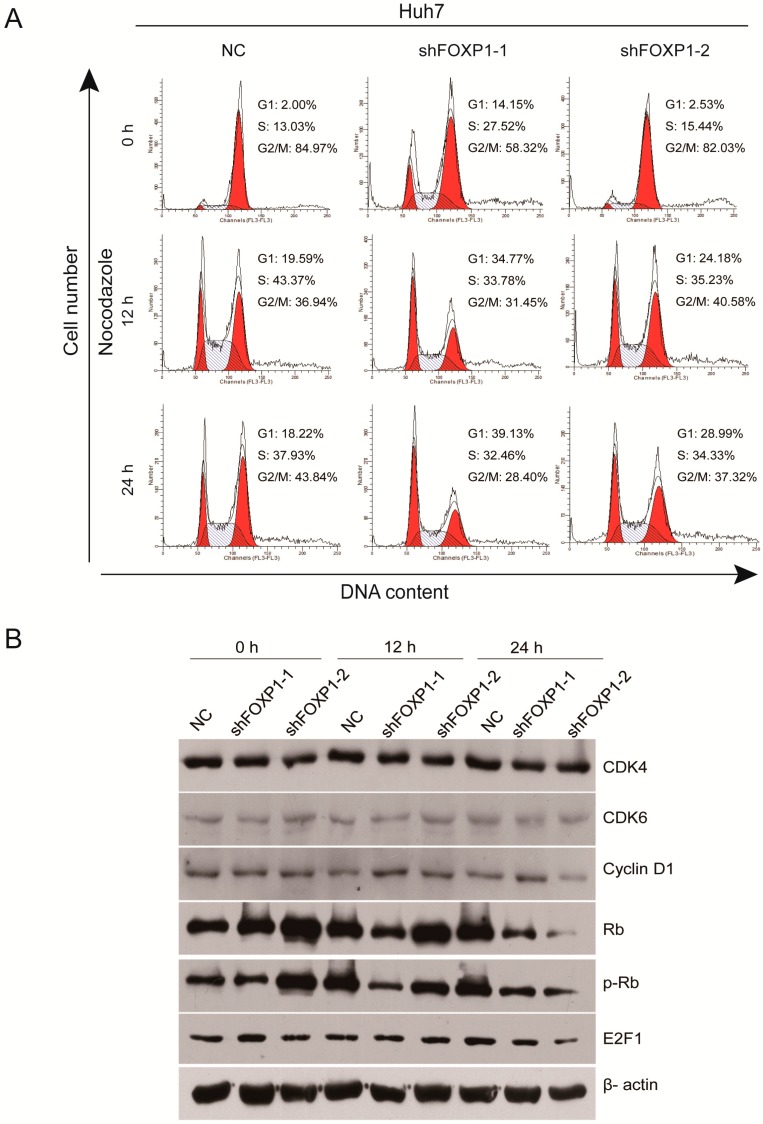
The effect of FOXP1 on G1/S phase transition and cell cycle-related proteins in HCC cells. (**A**) The cell cycle distribution of Huh7 cells that were stably transfected with either shFOXP1 or a scrambled sequence control; (**B**) Western blot analysis of the expression of G1/S phase transition-related proteins (CDK4, CDK6, cyclin D1, p-Rb, Rb, and E2F1) in Huh7 cells. β-actin was used as a loading control.

To further investigate the effect of FOXP1 on HCC cell growth, the cell cycle distribution among Huh7 cells was determined by flow cytometry. Nocodazole is a synthetic drug that has antimitotic and antitumor activities [23,24]. After treatment with 0.3 μM nocodazole for 24 h to synchronize cells at the G2/M boundary, the cells were collected at 0, 12, and 24 h. We found that downregulation of FOXP1 induced cell cycle arrest at the G1/S checkpoint. Furthermore, the accumulation of cells at G1/S phase persists for 12 and 24 h (Figure 4A, Table 1). We next detected the expression of key molecules that regulate the G1/S phase transition in lenti-shFOXP1 and lenti-control Huh7 cells; our results showed that the expression of total Rb, phosphorylated Rb, and E2F1 were markedly decreased at 24 h, although CDK4 and 6 and cyclin D1 did not show any noticeable changes (Figure 4B). These data indicated that the reduction of active Rb is the main contributor to G1/S phase arrest after knockdown of FOXP1.

## 3. Discussion

FOXP1 has been located to chromosome 3p14.1, a chromosomal locus that has been shown to be disrupted in a series of solid tumors [25]. Many studies have revealed that FOXP1 is aberrantly expressed in several types of cancers and its expression frequently has a significant correlation with aggressive malignant phenotypes of tumors and poor prognosis in patients [11,12,16,17,18,19,21,26,27]. Therefore, these findings suggest that FOXP1 indeed plays an important role in solid cancers and led us to investigate the role of FOXP1 in HCC.

In our study, we found that FOXP1 mRNA and protein levels are both higher in HCC tissues than in the adjacent non-tumorous tissues. Further analysis was conducted to identify the relationship between FOXP1 mRNA expression and the clinic-pathological parameters according to the TCGA. We observed that FOXP1 mRNA levels were significantly higher in HCC patients with higher serum α-fetoprotein (AFP) levels (Figure S1C; *p* = 0.0070). FOXP1 expression was remarkably correlated with HBV infection (Figure S1D; *p* = 0.0123) and liver cirrhosis (Figure S1E; *p* = 0.0008). Moreover, FOXP1 transcript levels were significantly upregulated in HCC with advanced histological grades (III and IV) compared to those with early grades (I and II) (Figure S1F; *p* < 0.0001). These results are in accordance with previous reports [21,28]. When endogenous FOXP1 was silenced after stable transfection of shRNA, the proliferative, migratory, and invasive abilities of Huh7 cells were significantly decreased (Figure S2A,B). These results are in accordance with a previous study [22]. The tumor xenograft models also confirmed that FOXP1 is a positive regulator of tumorigenicity in HCC.

It has been reported that FOXP1 participates in cell cycle regulation in many types of normal and cancerous tissues and organs. In hair follicle stem cells, overexpression of FOXP1 leads to cell cycle arrest. Furthermore, FOXP1 controls the expression of the cyclin-dependent kinase inhibitor p57KIP2 and the secreted growth factor Fgf18 [29]. FOXP1 regulates T cell quiescence and homeostasis in vivo, and FOXP1-deficient mature naive T cells gain an effector phenotype and function and proliferate in intact recipient mice [30]. Cell proliferation in FOXP1^−/−^ mouse hearts is aberrantly upregulated, but the trabecular myocardium in FOXP1^−/−^ embryos also exhibited increased p21 levels and decreased p27 levels, suggesting that cell cycle regulation is compromised in a complex manner in FOXP1^−/−^ mouse hearts [7].

Cell cycle dysregulation is a hallmark of tumor cells [31]. In neuroblastoma, there is an increased fraction of cells in the G0/G1 phase at the expense of the S phase population in FOXP1-expressing cells, and induction of FOXP1 delayed cell cycle progression and upregulated pro-apoptotic genes such as DIABLO, CDC42, and DAPK1, indicating the induction of the intrinsic pathway of apoptosis [32]. The G1/S checkpoint comprises cyclin D1 and E, CDK2, 4, and 6, p21 and p27 (CDK inhibitors), and Rb. In our study, we noticed that phosphorylated Rb levels were markedly decreased, while other proteins such as CDK4 and 6 and cyclin D1 did not significantly change after knockdown of FOXP1. This observation suggests that FOXP1 does not regulate the phosphorylation of Rb through CDK4 and 6. There may be a mechanism linking FOXP1 and phosphorylated Rb, which we will continue to explore and research.

## 4. Materials and Methods

### 4.1. Clinical Specimens and Expression Data Sets

Sixty-two HCC tissue specimens were obtained from patients who underwent surgical resection at the Qidong Liver Cancer Institute (Qidong, China). The patients were followed for at least five years. Among the collected specimens, 24 pairs of tumor and adjacent noncancerous liver tissues were chosen for FOXP1 protein detection. All of the procedures were approved by the China Ethical Review Committee. The FOXP1 mRNA expression data of 322 HCC patients were obtained from The Cancer Genome Atlas (TCGA, https://cancergenome.nih.gov/, updated to the end of 24 February 2015) database (hereinafter referred to as the TCGA cohort). Among them, 50 matched HCC and noncancerous liver samples were screened for FOXP1 mRNA levels, and the available clinical parameters were used for subsequent analysis.

### 4.2. Cell Lines and Cell Culture

The human HCC cell line Huh7 was obtained from the Riken Cell Bank (Tsukuba, Japan). MHCC-97L, MHCC-97H, and MHCC-LM3 cells were obtained from the Liver Cancer Institute of Zhongshan Hospital, Fudan University (Shanghai, China). Hep3B and PLC/PRF/5 cells were purchased from the American Type Culture Collection (ATCC) (Manassas, VA, USA). All of the cell lines used in this study were cultured in Dulbecco’s modified Eagle’s medium (DMEM) (Sigma-Aldrich, St. Louis, MO, USA) containing 10% fetal bovine serum (FBS) (HyClone, Logan, UT, USA) and then supplemented with 100 IU/mL penicillin G and 100 μg/mL streptomycin (Sigma-Aldrich). All of the cell lines were incubated at 37 °C in a humidified atmosphere with 5% CO_2_.

### 4.3. Quantitative Real-Time Polymerase Chain Reaction (qRT-PCR)

Total RNA was extracted from tissues and cells using TRIzol reagent (Invitrogen, Carlsbad, CA, USA). Reverse transcription was performed with PrimeScript RT Reagent Kit (Perfect Real Time) (TaKaRa Biotechnology, Dalian, China). PCR analysis was performed using specific primers for the FOXP1 gene: forward, 5′-CTTGCTCAAGGCATGATTCC-3′, and reverse, 5′-CCTTGGTTCGTCAGCCAGTA-3′. The expression levels were normalized to human GAPDH (glyceraldehyde-3-phosphate dehydrogenase) as an internal control: forward, 5′-AGAAGGCTGGGGCTCATTTG-3′ and reverse, 5′-TGAGAGCTGTCCATTGGTAGAG-3′.

### 4.4. Western Blot Analysis

Proteins extracted from cell lysates and tissue lysates were separated by using 10% SDS-PAGE and transferred onto nitrocellulose membranes. The membranes were incubated with primary antibody overnight at 4 °C and subsequently probed with horseradish peroxidase (HRP)-conjugated secondary antibodies (Table S1). The immunoreactive blots were visualized using an enhanced chemiluminescence reagent (Pierce, Rockford, IL, USA).

### 4.5. RNA Interference-Mediated Gene Silencing

The short hairpin RNAs targeting FOXP1 by lentivirus-based vector were designed by Genechem (Shanghai, China). The sequences were as follows: FOXP1-RNAi-1: GCGAAGATTTCCAATCATT; FOXP1-RNAi-2: CAGAAGTTAGACCACCATT and a scrambled sequence control (NC): TTCTCCGAACGTGTCACGT.

### 4.6. Colony Formation Assays

A total of 3000 cells per well were seeded in six-well plates and cultured at 37 °C. Approximately two weeks later, the cells were fixed with 10% formaldehyde and stained with Giemsa solution for 30 min. Each measurement was performed in triplicate.

### 4.7. MTT Assays

First, 3000 cells per well were seeded in 96-well plates and incubated for 24 h. Then, 10 μL of MTT reagent (5 mg/mL, Sigma-Aldrich) was added to each well and incubated for 4 h at 37 °C. Afterward, the formazan was sufficiently dissolved in 150 μL DMSO. After a five minute incubation, the absorbance at 570 nm of 100 μL of the dissolved formazan solution was measured by using an ELISA plate reader. Each measurement was performed in triplicate for seven consecutive days.

### 4.8. In Vitro Wound Healing Assays

The cells were seeded in six-well plates and grown to over 90% confluence overnight and then scratched within the confluent cell layer using the fine end of a 10 μL pipette tip. After three washes with serum-free DMEM, the cells were photographed under a 10× objective lens every 12 h on an inverted fluorescence microscope (Axiovert 200, HAL 100, Carl ZEISS, Oberkochen, Germany). The cells were continuously cultured in DMEM containing 2% FBS and 1 mmol/L thymidine after scratching.

### 4.9. In Vitro Invasion Assays

A total of 2 × 10^5^ cells was seeded into the upper chamber of a transwell (BD Biosciences, San Jose, CA, USA) in serum-free media, while the lower chamber of the transwell contained DMEM with 10% FBS. After 24 h of incubation, the cells in the upper chamber were removed. The cells were fixed with 10% formaldehyde for 30 min, stained with Giemsa solution and quantified.

### 4.10. Tumor Xenograft Models

HCC cells expressing stable shFOXP1 were orthotopically inoculated into the left hepatic lobe of six- to eight-week-old male BALB/c (nu/nu) nude mice, while HCC cells containing empty vector were used as a control. Approximately 3 × 10^6^ Huh7 cells were used for each mouse. Four weeks later, all of the mice were sacrificed. Immediately after euthanization, the xenograft tumors were weighed and fixed with 4% phosphate-buffered neutral formalin. Fixed tumor tissues were analyzed by Western blotting.

### 4.11. Flow Cytometry Analysis of Cell Cycle

For cell cycle analysis, cells were plated in six-well plates at 2 × 10^5^ cells per well for 24 h. The cells were pretreated with 0.3 μM nocodazole (Sigma-Aldrich) for 24 h to synchronize cells at the G2/M boundary. Cells were then harvested, washed twice with cold PBS, and fixed with cold 70% ethanol at −20 °C overnight. The cells were then washed twice with PBS and resuspended with 10 mg/mL RNase A, 400 mg/mL propidium iodide, and 0.1% Triton X in 1 mL PBS at 37 °C for 15 min. Cells were subsequently analyzed by flow cytometry, and DNA content was quantified using ModFit LT software (Verity Software House: Augusta, Topsham, ME, USA).

### 4.12. Statistical Analysis

The experimental data were presented as the mean ± SD and analyzed using Student’s *t*-test. A *p* value less than 0.05 was considered statistically significant.

## 5. Conclusions

In conclusion, our study emphasizes that downregulation of FOXP1 is able to inhibit cell proliferation in hepatocellular carcinoma via inducing G1/S phase cell cycle arrest, in which the decrease of phosphorylated Rb is the main contributor. The functional details of FOXP1 in human primary HCC need to be explored.

## Figures and Tables

**Table 1:** Cell cycle distribution of Huh7 cells after transfecting lenti-control and lenti-shFOXP1.

Time	Cell Cycle	NC^#^ (%) shFOXP1-1 (%) shFOXP1-2 (%)		
0 h	G1	2.22 ± 0.21	14.7 ± 0.57 ***	2.02 ± 0.44
	S	14.55 ± 1.55	27.64 ± 0.40 **	15.85 ± 0.86
	G2/M	83.22 ± 1.68	57.65 ± 0.90 **	82.13 ± 0.75
12 h	G1	21.96 ± 2.97	34.70 ± 0.91 *	24.02 ± 1.14
	S	43.52 ± 2.18	35.21 ± 1.25 **	36.92 ± 1.53 *
	G2/M	34.49 ± 2.12	30.08 ± 1.56 *	39.06 ± 2.07
24 h	G1	20.37 ± 1.86	37.73 ± 1.78 ***	28.3 ± 0.75 **
	S	37.55 ± 1.88	32.28 ± 0.41 *	35.16 ± 1.07
	G2/M	42.07 ± 2.42	29.99 ± 1.65 **	36.76 ± 0.59

NC^#^, negtive control shRNA. Data are mean ± SD of three independent experiments. Student *t* test, vs. the control group. * *p* < 0.05, ** *p* < 0.01, *** *p* < 0.001.

## Supplementary Materials

Supplementary materials can be found at www.mdpi.com/1422-0067/17/9/1501/s1.
