# Supplementary material for: Downregulation of FOXP1 Inhibits Cell Proliferation in Hepatocellular Carcinoma by Inducing G1/S Phase Cell Cycle Arrest
Source: Int J Mol Sci. 2016 Sep 8;17(9):1501. doi: 10.3390/ijms17091501 (PMC5037778; doi:10.3390/ijms17091501)
Supplement: Supplementary file 1 [file ijms-17-01501-s001.pdf]

Supplementary Materials: Downregulation of FOXP1 Inhibits Cell Proliferation in Hepatocellular Carcinoma by Inducing G1/S Phase Cell Cycle Arrest

Xin Wang, Ji Sun, Meiling Cui, Fangyu Zhao, Chao Ge, Taoyang Chen, Ming Yao and Jinjun Li

Table S1. Antibodies used for Western blot.

Antibody	Host	Dilution	Company
FOXP1	Rabbit polyclonal	1:500	Cell Signaling Technology
CDK4	Rabbit polyclonal	1:200	Santa Cruz
CDK6	Mouse mAb IgG1	1:100	Santa Cruz
Cyclin D1	Goat polyclonal	1:200	R&D
p-Rb	Rabbit polyclonal	1:500	Cell Signaling Technology
Rb	Mouse mAb IgG2a	1:500	Cell Signaling Technology
E2F1	Mouse mAb IgG2a	1:200	Santa Cruz
β-actin	Mouse mAb	1:10,000	Sigma-Aldrich

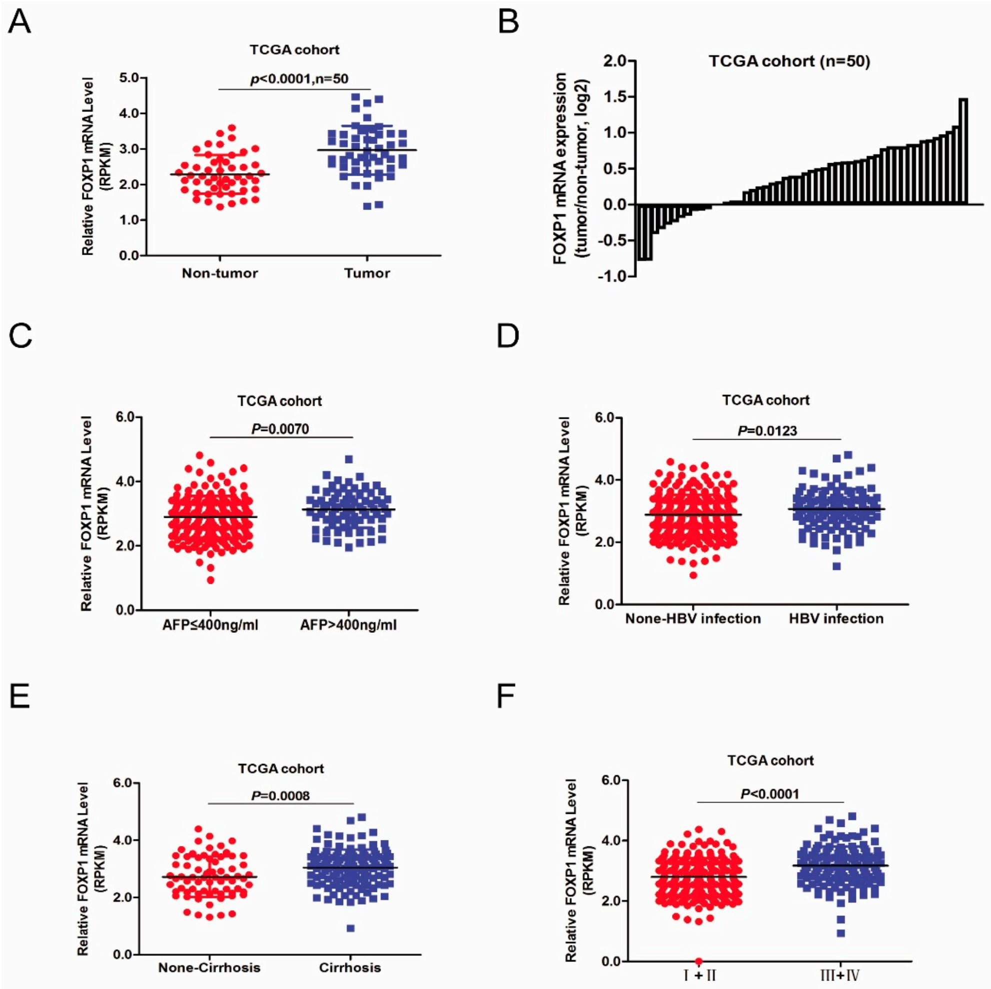

Figure S1. FOXP1 expression in HCC clinical samples and its relationship with clinicopathological parameters of HCC patients according to TCGA. (**A**) FOXP1 mRNA level in the TCGA cohort; (**B**) The fold change of FOXP1 level in paired tumor/non-tumorous tissues of the TCGA cohort; (**C–F**) Relationship between FOXP1 mRNA level and serum AFP level (**C**); HBV infection (**D**); liver cirrhosis (**E**) and histological grades (**F**). In the TCGA cohort, data are depicted as RPKM (read per kilo bases per million reads).

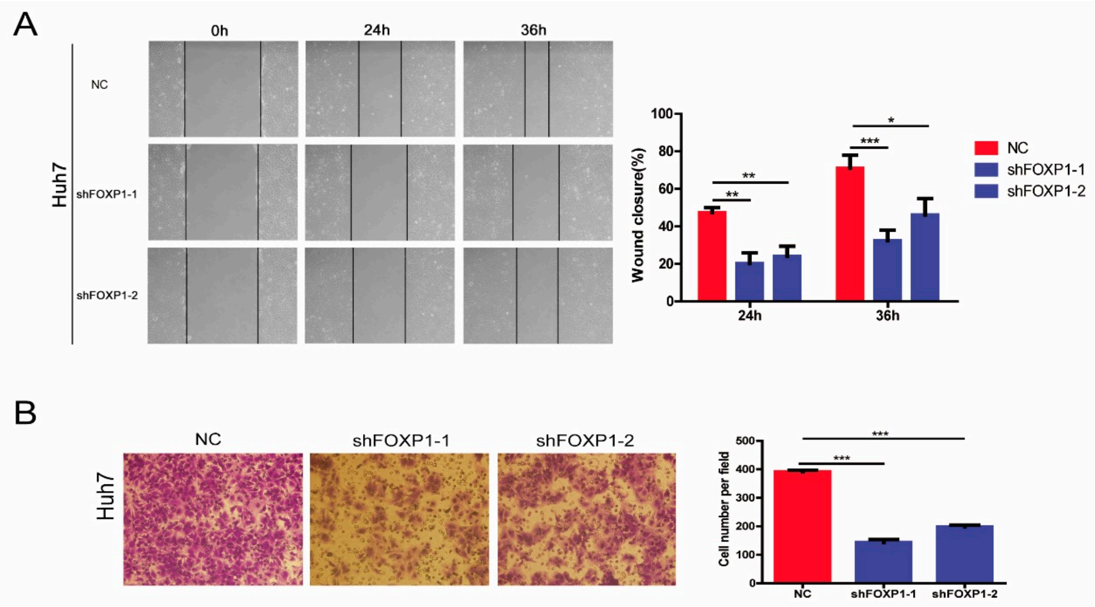

Figure S2. The effect of FOXP1 on the migration and invasion ability of HCC cells in vitro. (**A**) Wound healing assays for Huh7 cells that were stably transfected with shFOXP1 or NC; (**B**) Transwell invasion assays for Huh7 cells that were stably transfected with shFOXP1 or NC. *** $p < 0.001$, ** $p < 0.01$, * $p < 0.05$.
